# Long-term sickness absence trajectories among ageing municipal employees – the contribution of social and health-related factors

**DOI:** 10.1186/s12889-023-16345-9

**Published:** 2023-07-26

**Authors:** Johanna Suur-Uski, Olli Pietiläinen, Aino Salonsalmi, Johanna Pekkala, Pi Fagerlund, Ossi Rahkonen, Tea Lallukka

**Affiliations:** Department of Public Health, University of Helsinki, Tukholmankatu 8B, P.O. Box 20, Helsinki, 00014 Finland

**Keywords:** Sickness absence, Sick leave, Trajectories, Cohort study, Ageing, Health behaviour, Work arrangements

## Abstract

**Background:**

The ageing work force is heterogeneous, following distinct development in work ability. This study aims to identify trajectories of long-term sickness absence (SA) in later careers and to examine potentially modifiable factors associated with the development of SA.

**Methods:**

Data comprised of municipal employees of the city of Helsinki aged 50–60 years during 2004–2018 (N = 4729, 80% women). The developmental trajectories of long-term (> 10 working days) SA were examined with Group-based trajectory modelling (GBTM) using SA records of the Social Insurance Institution of Finland during 2004–2018. All-cause and diagnosis-specific (mental disorder– and musculoskeletal disease–related) SA days were analysed. The association of social and health-related factors with trajectory membership was examined using multinomial logistic regression (odds ratios and 95% confidence intervals).

**Results:**

A model with three trajectories was selected for both all-cause and diagnosis-specific SA. Regarding all-cause long-term SA trajectories, 42% had no long-term SA, 46% had low levels of SA, and 12% had a high rate of SA during follow-up. Lower occupational class, reporting smoking, overweight or obesity, moderate or low leisure-time physical activity, and sleep problems were associated with a higher likelihood of belonging to the trajectory with a high rate of SA in both all-cause and diagnosis-specific models.

**Conclusions:**

Most ageing employees have no or little long-term SA. Modifiable factors associated with trajectories with more SA could be targeted when designing and timing interventions in occupational healthcare.

**Supplementary Information:**

The online version contains supplementary material available at 10.1186/s12889-023-16345-9.

## Background

The share of older employees has risen rapidly, and employees 55 or older comprise a fifth of the employed population in the European Union (EU) [1]. Whilst sickness absence (SA) rates typically increase with age, [2, 3] the ageing work force is heterogeneous, following different trajectories of work ability. Relatively little is known about the development of SA in later career considering the recent shift towards a more mature work force.

SA is a well-established indicator of poor health, and long-term SA (> 10 working days) has been linked with diseases [2] and mortality [4]. In general, good health supports work ability and older employees have more chronic diseases [5]. In recent years, mental disorder–related SA has increased [6, 7] and is now one of the main causes for long-term SA [8]. Among ageing employees in Finland, SA due to mental disorders is becoming more frequent and musculoskeletal disease–related SA is decreasing [6]. This trend is more prominent among women.

Most previous studies on SA in later career are variable-oriented and only a few previous studies utilise person-based methods. To our knowledge, three previous studies have explored the developmental trajectories of all-cause SA in later careers. These include a Finnish study using data from the 1980s, [9] a comparative Finnish study on SA in the private versus municipal sectors, [10] and a Spanish study on both young and ageing employees [11]. All these studies identified three trajectories and a small subgroup with worsening work ability. Apart from one, [9] these studies lack information on health behaviour and thus are unable to identify behavioural factors for future interventions.

Besides health, socioeconomic and psychosocial factors, work arrangements, and even pension systems are associated with SA [12, 13]. It has been established that SA is more common among women and among those in lower occupational classes [2]. Similarly, health- [14–19] and work-related factors [19–23] are linked to SA. Many previous studies on work-related factors have focused on healthcare personnel [19, 21, 23] and register-based studies often lack information about health behaviour and working conditions [24].

Given the challenge presented to society by the change in labour market composition, more information is needed on how employees with chronic disabilities cope in the work force and how their work ability can be supported. Specifically, characterisation of the heterogeneity in the ageing workforce might present new grounds for targeted support for employees.

In this study, we examined the developmental trajectories of all-cause and diagnosis-specific long-term SA in a municipal employee cohort comprising various occupational groups. Furthermore, we elucidated the characteristics that are associated with trajectory membership, including socioeconomic factors, health behaviours, and work arrangements.

## Methods

Our study is a part of Helsinki Health Study, an employee cohort study that focuses on health and workability of the employees of the city of Helsinki, Finland. The baseline sample included employees turning 40, 45, 50, 55, or 60 during the years 2000–2002 [25]. In all, 8960 participants (response rate 67%) replied to the phase 1 questionnaire. Follow-up questionnaires were mailed in 2007 (phase 2, response rate 83% N = 7332), 2012 (phase 3, response rate 79% N = 6814), and 2017 (phase 4, response rate 82% N = 6832). Approximately 80% of the cohort were women, corresponding to what is typical in the municipal sector in Finland.

### Participant selection

For our study, we subtracted those who replied to baseline questionnaire and gave permission for register linkage (n = 6486). We included those aged 50–60 years during 2004–2018 (n = 801 excluded). Participants SA data was not considered after retirement. If the participant retired during year 2004 they were excluded from the follow up totally but if they retired in 2016 they could participate to the model until 2015 (excluded n = 113 participants). The analysis method, required multiple longitudinal time points, hence three years of information on SA was set as the minimum requirement for eligibility with either three successive or three separate years (excluded n = 711). If a person had missing years during the follow-up those years were considered not available in the analysis. After exclusions, the data included 4729 participants. Follow-up started in 2004 with a mean follow-up time of 8.7 years.

### Sickness absence data

SA data were derived from the Social Insurance Institution of Finland (SII) sickness allowance register. In Finland, all residents aged 16–67 years are eligible for sickness allowance as compensation for work disability. For the employee, the first ten working days (calendar days excluding mid-week holidays and Sundays) are compensated by the employer. After that, the allowance is granted by the SII. Sickness allowance granted by the SII always requires a medical certificate. The SA data retrieved from SII included annual information on long-term SA in 2004–2018 and a diagnostic code for each episode based on the International Classification of Diseases (ICD-10). We extracted all-cause SA and the two most common diagnosis-specific SA groups: mental disorder–related (MD) long-term SA (ICD-10 codes F00–F99) and musculoskeletal disease–related (MSD) long-term SA (ICD-10 codes M00–M99). Sickness absence days per year were transformed to account for months per year as follows: 0–13 days = 0 months, 14–29 days = 1 month, 30–59 days = 2 months etc., and 12 months was defined as 330 or more SA days. Information on retirement was derived from the Finnish Centre for Pensions. The two most common diagnostic groups for disability pension in Finland are musculoskeletal diseases and mental disorders. The register information was linked to the questionnaire data.

### Social factors

Gender and age were derived from the questionnaires. Occupational class was derived from the City of Helsinki personnel register and classified into four categories: professionals and managers (teachers, doctors, managers with subordinates), semi-professionals (nurses, foremen), routine non-manual employees (child minders, assistant nurses), and manual workers (work in transport, cleaners) [26]. Marital status was derived from the questionnaires and dichotomised as cohabiting (married/cohabiting) or non-cohabiting (divorced/widowed/single).

### Work-related factors

Work arrangements and work—home satisfaction were derived from the questionnaires. Work was categorised as regular daytime work (including daytime work with on call nightshifts) or shift work (including shift work with regular night shifts, regular night work, and work type other). Self-reported working hours were counted as the mean of reported working hours over time.

Work—home satisfaction was estimated by question ‘How satisfied are you with combining paid work and family?’ [27] Replying satisfied or somewhat satisfied was classified as work—home satisfaction ‘Good’ and replying somewhat not satisfied or not satisfied as ‘Poor’.

### Health-related factors

Self-reported smoking, binge drinking, weight, sleep problems, and leisure time physical activity were examined as health-related behavioural factors. Smoking was divided into non-smoking (including ex-smoking) and smoking (current smoking, daily or occasionally). Binge drinking was indicated by drinking six or more units of alcohol at least once a week on a single occasion. Body mass index (BMI) (from self-reported weight and height) was categorised into healthy weight (BMI < 25), overweight (25 ≤ BMI < 30), and obesity (BMI ≥ 30). Sleep was measured on a four-item version of the Jenkins questionnaire to indicate sleep problems [28, 29]. Having at least 1 of the 4 symptoms occurring at least 15 times within the past 4 weeks was classified as having sleep problems. Leisure time physical activity was estimated using the metabolic equivalent (MET) index [30] and divided into three groups: low (< 14 MET-hours/week), intermediate (≥ 14 MET-hours/week with only moderate activity) and high (≥ 14 MET-hours/week including vigorous activity). For each participant, the covariates were derived from the questionnaires using the mode [31].

### Statistical methods

**Fig. 1:**
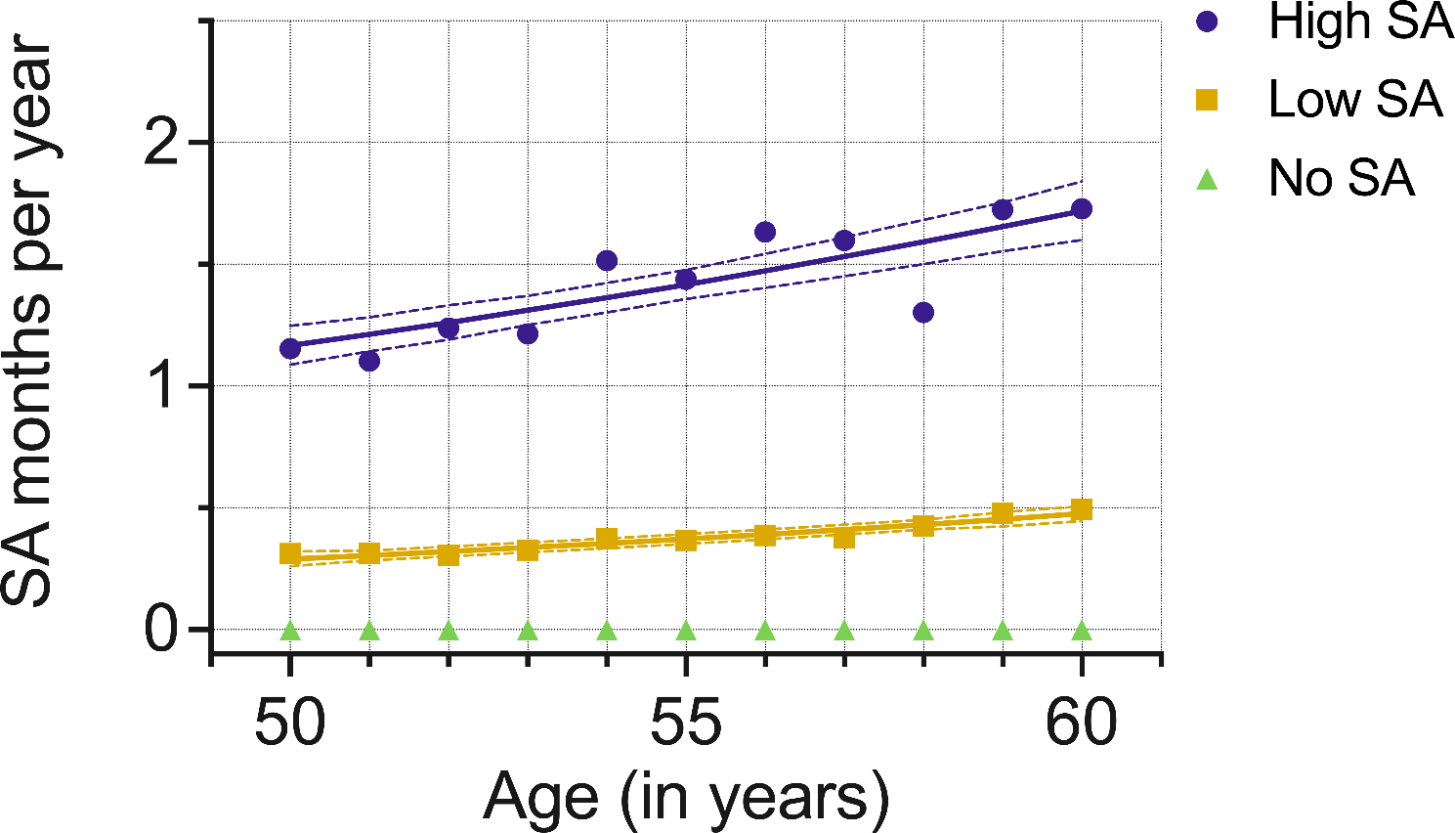
All-cause long-term (> 10 working days) sickness absence (SA) trajectories’ averages and estimates with 95% confidence intervals identified in the group-based trajectory modelling (GBTM) (N = 2747). Follow-up between 50–60 years of age. Sickness absence by age from the model. The ‘No SA’ trajectory group (directly observed from the data) was added after GBTM (N = 4729). ‘No SA’ (42% n = 1982), ‘Low SA’ (46%, n = 2183), and ‘High SA’ (12%, n = 564)

**Fig. 2:**
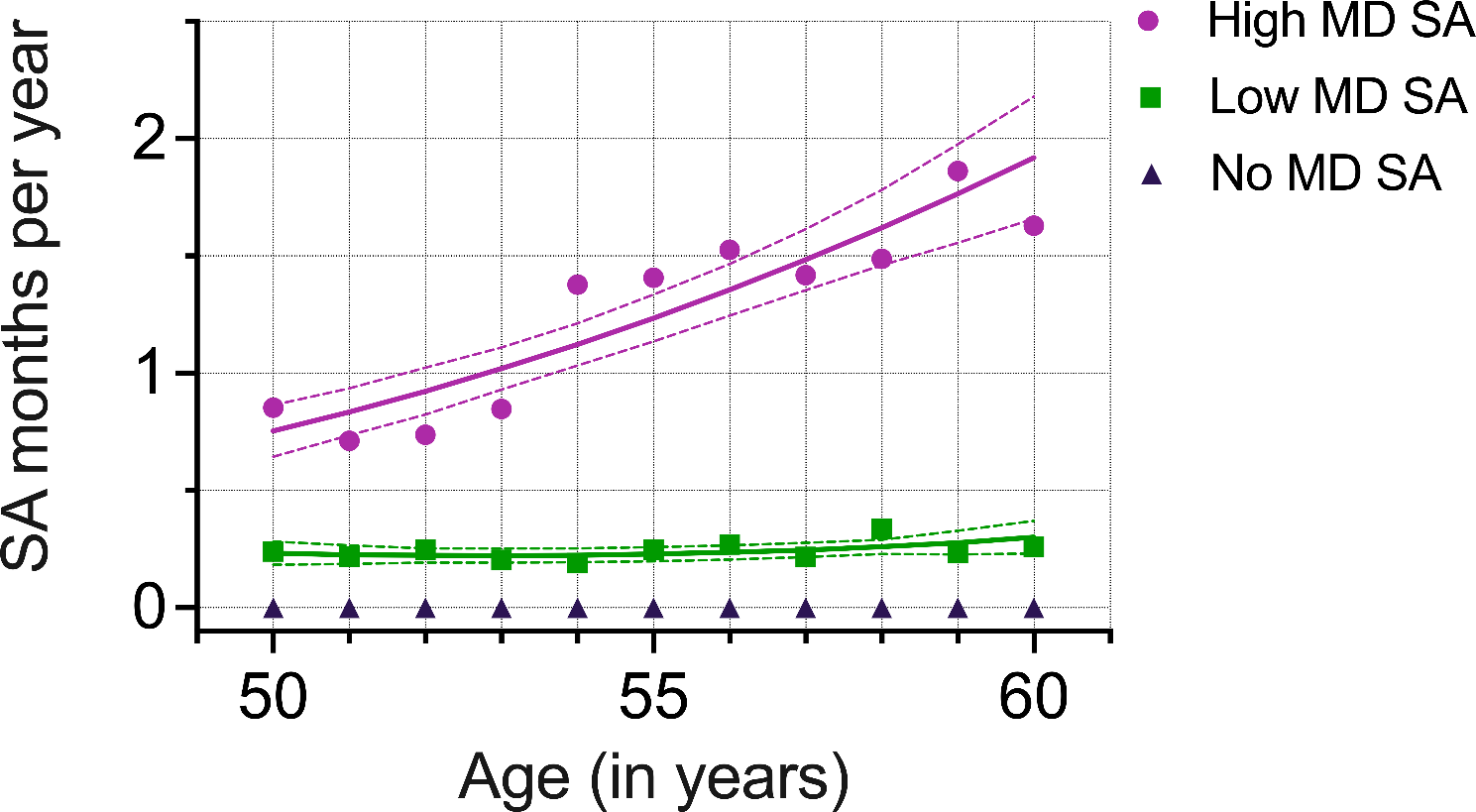
Mental disorder–related long-term (> 10 working days) sickness absence (SA) trajectories’ averages and estimates with 95% confidence intervals identified in the group-based trajectory modelling (GBTM) (N = 697). Follow-up between 50–60 years of age. Fitted SA rate by age from the GBTM model. The ‘No MD SA’ trajectory group was added after GBTM (N = 4729). ‘‘No MD SA’ (85%, n = 4032), ‘Low MD SA’ (10%, n = 482) and ‘High MD SA’ (5%, n = 215); MD = Mental disorder

**Fig. 3:**
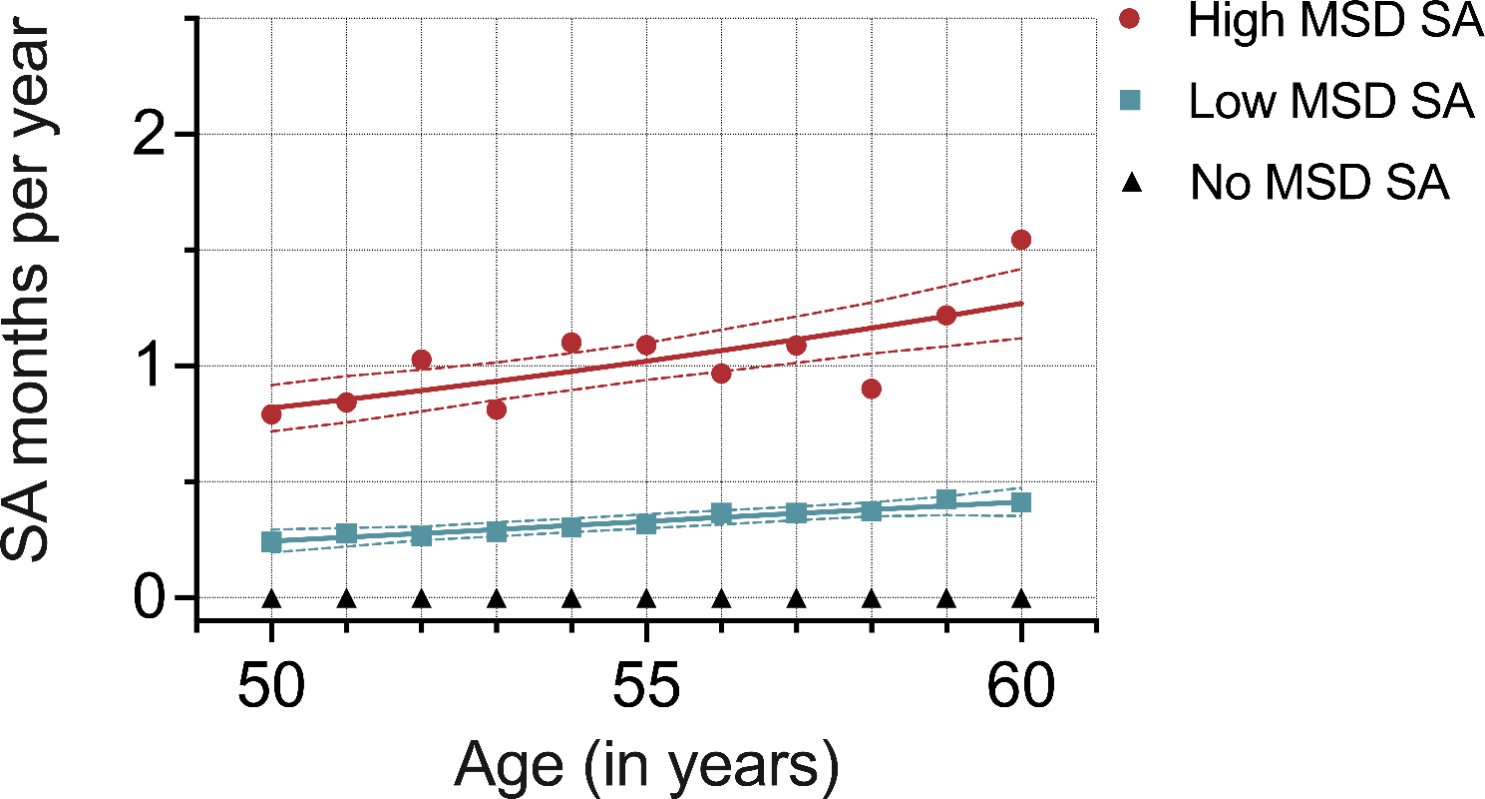
Musculoskeletal disease–related long-term (> 10 working days) sickness absence (SA) trajectories’ averages and estimates with 95% confidence intervals identified in the group-based trajectory modelling (GBTM) (n = 1273). Follow-up between 50–60 years of age. Fitted SA rate by age from the model. The ‘No MSD SA’ trajectory group was added after GBTM (N = 4729). ‘No MSD SA’ (73%, n = 3456), ‘Low MSD SA’ (20%, n = 930), ‘High MSD SA’ (7%, n = 343); MSD = Musculoskeletal disease

The development of long-term SA months was modelled for all-cause, mental disorder–related, and musculoskeletal disease–related SA by age (from 50 to 60 years). Trajectories were estimated using Group-based trajectory modelling, GBTM, which is used to identify distinct groups of the study population with similar developmental trajectories over time [32]. The annual number of SA months was used as an outcome with a zero-inflated Poisson distribution given the large amount of zero outcomes. The number of trajectory groups was selected based on the following criteria: Bayesian Information Criterion (BIC), Akaike Information Criterion (AIC), posterior probabilities of group membership, identifiability of the groups, and preference for plausible models that produced groups with no fewer than 5% of the participants. The trajectory analyses were computed with the software Stata using the procedure ‘Traj’. We accounted for non-random dropout in the models [33]. The syntax for all-cause model, criteria for choosing all-cause model and individual trajectories, and dropout probabilities for all of the models are presented in supplementary material (Supplementary material Table 1; Figs. 1–6).

Post trajectory analysis were computed in R. Cross-tabulations and Chi2-tests were performed (Supplementary Tables 2–4). Multinomial logistic regression models with odds ratios (OR) and 95% confidence intervals (CI) were fitted to examine how socioeconomic and work- and health-related factors were associated with trajectory membership using the package ‘nnet’.

We primarily analysed the trajectories for men separately, but they produced similar results as the models pooling women and men (data not shown). Due to the small number of men, we then carried out the analysis with genders pooled and conducted the multinomial logistic regression models adjusting for gender.

### Patient and public involvement

Patients or the public were not directly involved in designing or conducting the study. The results of Helsinki Health Study are regularly reported to the City of Helsinki to actively disseminate our results to the workplaces from where the information is gathered.

## Results

Most of the participants had regular daytime work (80%) (Table 1, supplementary material). The two largest occupational classes were professionals and managers and routine non-manual workers. Two-thirds were cohabiting. One-fifth reported smoking and a tenth binge drinking. Healthy BMI was reported by half, overweight by a third and obesity by less than a fifth. One-fifth reported sleep problems. Almost half (44%) were not satisfied with their work—home balance.

### Long-term all-cause sickness absence trajectories

A ‘No SA’ group was extracted (n = 1982) first since this trajectory could be observed directly from the data(24). This left 2747 participants for the GBTM, in which a model with two latent groups was selected and combined to the ‘No SA’ group for further analysis (variable-oriented step). The final model with three trajectories was: ‘No SA’ (42% n = 1982), ‘Low SA’ (46%, n = 2183), and ‘High SA’ (12%, n = 564)(Fig. 1). The ‘Low SA’ trajectory was around half a month and slightly increased during follow-up (Fig. 1). The ‘High SA’ trajectory SA rose from around one month to around 1,75 months per year during the follow-up.

Table 1 presents the all-cause SA trajectories multinomial logistic regression model adjusted for start year in the cohort, socioeconomic, work-related, and health-related factors. ‘No SA’ trajectory was used as comparison. Lower occupational class, reporting shift work, smoking, overweight, obesity, or sleep problems were associated with a higher (ORs ranging from 1.16 to 3.78) and male gender with a lower likelihood of belonging to the ‘Low SA’ and ‘High SA’ trajectories compared to ‘No SA’ trajectory (ORs ranging from 0.51 to 0.69). Those reporting not satisfied with work–home interaction, binge drinking weekly or more often, and moderate or low leisure-time physical activity had a higher likelihood of being assigned to ‘High SA’ trajectory compared to ‘No SA’ (ORs ranging from 1.28 to 1.55). There was no association for marital status.

**Table 1:** All-cause long-term (> 10 working days) sickness absence (SA) trajectories: The associations between socioeconomic, work-related, and health-related factors with SA trajectory membership. Multinomial logistic regression model with odds ratios (ORs) and their 95% confidence intervals (CIs)

All-cause SA		
	OR (95% CI)	
	Reference group: No SA trajectory (N = 1982)	
	Low SA (N = 2183)	High SA (N = 564)
Gender (Ref. Women)		
Men	**0.69 (0.59–0.80)**	**0.51 (0.46–0.56)**
Work arrangements (Ref. Non-shift work)		
Shift work	**1.18 (1.04–1.35)**	**1.18 (1.08–1.29)**
Working hours (weakly mean)	0.99 (0.98–1.00)	**0.98 (0.97–1.00)**
Work–home satisfaction (Ref. Satisfied)		
Not satisfied	1.08 (0.95–1.22)	**1.35 (1.13–1.60)**
Occupational class (Ref. Professionals and managers)		
Semi-professionals	**1.27 (1.13–1.43)**	**1.73 (1.56–1.92)**
Routine non-manual workers	**1.67 (1.49–1.88)**	**2.41 (2.09–2.79)**
Manual workers	**2.11(1.92–2.32)**	**3.78 (3.40–4.21)**
Marital status (Ref. Cohabiting)		
Non-cohabiting	1.06 (0.95–1.22)	0.98 (0.88–1.09)
Smoking (Ref. Never or quitted)		
Daily or occasionally	**1.35 (1.18–1.55)**	**1.88 (1.67–2.11)**
Binge drinking (six or more units) (Ref. Rarely or never)		
Weekly or more often	1.06 (0.89–1.25)	**1.28 (1.17–1.40)**
BMI (Ref. Healthy weight (BMI < 25))		
Overweight (25 ≤ BMI < 30)	**1.16 (1.02–1.33)**	**1.47 (1.25–1.73)**
Obesity (BMI ≥ 30)	**1.45 (1.24–1.70)**	**1.86 (1.25–1.73)**
Leisure-time physical activity (Ref. High)		
Moderate	1.05 (0.92–1.20)	**1.43 (1.25–1.64)**
Low	0.89 (0.76–1.05)	**1.55 (1.39–1.73)**
Sleep problems (Ref. No)		
Yes	**1.29 (1.14–1.48)**	**2.00 (1.83–2.18)**

Odds Ratios (OR) with 95% confidence intervals. Model adjusted for: start year in the follow-up, gender, work–related factors, occupational class, marital status and health behaviour, SA = sickness absence

### Diagnosis-specific long-term sickness absence trajectories

For mental disorder–related SA, a ‘No MD SA’ trajectory with no absences was extracted first (n = 4032), leaving 697 participants for GBTM. The final model with three mental disorder–related SA trajectories was: ‘No MD SA’ (85%, n = 4032), ‘Low MD SA’ (10%, n = 482) and ‘High MD SA’ (5%, n = 215)(Fig. 2). Compared to all-cause model, a smaller percentage of people belonged to the ‘High MD SA’ trajectory, in which the SA rate rose more drastically, rising from less than 1 month to 2 months. The ‘Low MD’ SA trajectory remained stable around 0.25 months.

In the mental disorder–related multinomial regression model, sleep problems (ORs ranging from 1.55 to 2.15) were associated with a higher likelihood and male gender (ORs ranging from 0.49 to 0.80) with a lower likelihood of being assigned to the trajectories ‘Low MD SA’ and ‘High MD SA’ compared to ‘No MD SA’ when adjusted for start year in the cohort, socioeconomic, work-related, and health-related factors (Table 2). Lower occupational class, reporting smoking, and leisure time physical inactivity were positively associated with the ‘High MD SA’ trajectory (ORs ranging from 1.43 to 2.05), whereas, working hours were negatively associated with the ‘High MD SA’ trajectory compared to ‘No MD SA’ trajectory.

**Table 2:** Mental disorder–related long-term (> 10 working days) sickness absence (SA) trajectories: The associations between socioeconomic, work-related, and health-related factors with SA trajectory membership. Multinomial logistic regression model with odds ratios (ORs) and their 95% confidence intervals (CIs); MD = Mental disorder

Mental disorder–related SA		
	OR (95% CI)	
	Reference group: No MD SA trajectory (4032)	
	Low MD SA (N = 482)	High MD SA (N = 215)
Gender (Ref. Women)		
Men	**0.49 (0.36–0.66)**	**0.8 (0.71–0.91)**
Work arrangements (Ref. Non-shift work)		
Shift work	1.02 (0.81–1.29)	0.97 (0.70–1.36)
Working hours (weakly mean)	0.98 (0.97–1.01)	**0.97 (0.95–1.00)**
Work–home satisfaction (Ref. Satisfied)		
Not satisfied	**1.40 (1.15–1.71)**	1.27 (0.95–1.70)
Occupational class (Ref. Professionals and managers)		
Semi-professionals	0.99 (0.80–1.22)	**1.64 (1.26–2.12)**
Routine non-manual workers	1.12 (0.94–1.32)	**1.74 (1.40–2.14)**
Manual workers	0.96 (0.80–1.14)	**1.67 (1.34–2.07)**
Marital status (Ref. Cohabiting)		
Non-cohabiting	**1.34 (1.09–1.64)**	0.99 (0.73–1.34)
Smoking (Ref. Never or quitted)		
Daily or occasionally	1.10 (0.86–1.40)	**1.57 (1.18–2.09)**
Binge drinking (six or more units) (Ref. Rarely or never)		
Weekly or more often	**1.36 (1.00–1.85)**	1.1 (0.98–1.25)
BMI (Ref. Healthy weight (BMI < 25))		
Overweight (25 ≤ BMI < 30)	0.98 (0.79–1.22)	**1.43 (1.16–1.75)**
Obesity (BMI ≥ 30)	1.04 (0.79–1.36)	**1.73 (1.47–2.04)**
Leisure-time physical activity (Ref. High)		
Moderate	0.98 (0.78–1.23)	**1.60 (1.34–1.91)**
Low	1.12 (0.85–1.46)	**2.05 (1.76–2.39)**
Sleep problems (Ref. No)		
Yes	**1.55 (1.24–1.94)**	**2.15 (1.59–2.91)**

Odds Ratios (OR) with 95% confidence intervals. Model adjusted for: start year in the follow-up, gender, work–related factors, occupational class, marital status and health behaviour, SA = sickness absence

For musculoskeletal disease–related SA, a ‘No MSD SA’ trajectory with no SA spells was extracted first (n = 3456), leaving 1273 participants for GBTM. A model with two latent groups was selected and combined to the ‘No MSD SA’ group. The final model for musculoskeletal diseases had likewise three trajectories: ‘No MSD SA’ (73%, n = 3456), ‘Low MSD SA’ (20%, n = 930), ‘High MSD SA’ (7%, n = 343)(Fig. 3). The ‘Low MSD SA’ trajectory increased from quarter to half a month and the ‘High MSD SA’ increased from less than one month to 1.25 months.

In the musculoskeletal disorder–related multinomial regression model lower occupational class, smoking, overweight, obesity, and sleep problems were associated with a higher (ORs ranging from 1.16 to 3.75), and male gender with a lower (ORs ranging from 0.40 to 0.79) likelihood of being assigned to either trajectory with SA compared to ‘No SA’ trajectory (Table 3). Reporting leisure time physical inactivity was associated with a higher likelihood of belonging to the ‘High MSD SA’ trajectory compared to ‘No MSD SA’ trajectory (ORs ranging from 1.57 to 1.79). Shift work associated with a higher (OR 1.26, 95% CI 1.06 to 1.50) and binger drinking with a lower (OR 0.76, 95% CI 0.59 to 0.98) likelihood of being assigned to ‘Low MSD SA’ trajectory compared to ‘No MSD SA’.

**Table 3:** Musculoskeletal disease–related long-term (> 10 working days) sickness absence (SA) trajectories: The associations between socioeconomic, work-related, and health-related factors with SA trajectory membership. Multinomial logistic regression model with odds ratios (ORs) and their 95% confidence intervals (CIs); MSD = Musculoskeletal disease

Musculoskeletal disease–related SA		
	OR (95% CI)	
	Reference group: No MSD SA trajectory (N = 3456)	
	Low MSD SA (N = 930)	High MSD SA (N = 343)
Gender (Ref. Women)		
Men	**0.79 (0.64–0.98)**	**0.40 (0.38–0.43)**
Work arrangements (Ref. Non-shift work)		
Shift work	**1.26 (1.06–1.50)**	1.13 (0.86–1.47)
Working hours (weakly mean)	0.99 (0.98–1.01)	0.99 (0.97–1.01)
Work–home satisfaction (Ref. Satisfied)		
Not satisfied	0.91 (0.78–1.06)	1.25 (0.99–1.59)
Occupational class (Ref. Professionals and managers)		
Semi-professionals	**1.46 (1.25–1.70)**	**1.64 (1.33–2.03)**
Routine non-manual workers	**2.30 (2.03–2.60)**	**2.88 (2.42–3.43)**
Manual workers	**3.04 (2.65–3.48)**	**3.75(3.09–4.55)**
Marital status (Ref. Cohabiting)		
Non-cohabiting	0.96 (0.81–1.14)	0.92 (0.72–1.18)
Smoking (Ref. Never or quitted)		
Daily or occasionally	**1.54 (1.28–1.85)**	**1.84 (1.45–2.33)**
Binge drinking (six or more units) (Ref. Rarely or never)		
Weekly or more often	**0.76 (0.59–0.98)**	1.07 (0.96–1.18)
BMI (Ref. Healthy weight (BMI < 25))		
Overweight (25 ≤ BMI < 30)	**1.19 (1.00–1.41)**	**1.53 (1.28–1.83)**
Obesity (BMI ≥ 30)	**1.53 (1.25–1.87)**	**1.75 (1.54–2.00)**
Leisure-time physical activity (Ref. High)		
Moderate	1.10 (0.92–1.30)	**1.57 (1.35–1.83)**
Low	0.95 (0.77–1.18)	**1.79 (1.57–2.03)**
Sleep problems (Ref. No)		
Yes	**1.16 (1.03–1.50)**	**1.93 (1.50–2.49)**

Odds Ratios (OR) with 95% confidence intervals. Model adjusted for: start year in the follow-up, gender, work–related factors, occupational class, marital status and health behaviour, SA = sickness absence

## Discussion

We studied the developmental patterns of long-term SA among ageing municipal employees. A three-trajectory model was selected for all-cause and diagnosis-specific SA. Most employees had a higher likelihood of belonging to the trajectories with no or low SA. All the models possessed an intermediate trajectory with a slight increase in SA at later ages and a smaller trajectory with a constantly high rate of SA that increased with age. In the all-cause model, women, lower occupational class, shift work, and unhealthy health behaviours were associated with a higher likelihood of being assigned to the two trajectories with SA. In the diagnosis-specific models, women, lower occupational class, smoking, leisure time physical inactivity, and sleep problems were associated with being assigned to the trajectory with the highest rate of SA, whereas the results varied for work-related factors.

Previous studies examining trajectories of SA among ageing employees have identified a trajectory with a high and increasing rate of SA, [9–11] comprising around 10% of the participants. A Spanish study, including those with at least one SA spell, identified a decreasing trajectory in addition to a low and a high trajectory, however, comprising only 4% of the participants [11]. The study, however, also included shorter SA spells that might not be as strongly linked to diseases and functioning. Previous studies have modelled SA by calendar years and with shorter follow-up times than this study, ranging from three to six years. We modelled the development of long-term SA by age with a longer follow-up and included a wide variety of social and health- and work-related factors.

Long-term SA has been associated with recurring absence [34]. In line with this, we found no trajectory with a declining trend. The highest increase during follow-up was observed in the mental disorder–related SA model, where the trajectory ‘High MD SA’ started at a lower rate than all-cause model, increased throughout the follow-up, and reached a higher level (2 months) compared to the other models. In the musculoskeletal disease model, the ‘High MSD SA’ started from the same level as mental disorder–related SA model but did not increase equally much. But the ‘Low MSD SA’ trajectory increased more with age compared to the mental disorder-related ‘Low MD SA’ trajectory. These observations might reflect the disease prognosis. Mental disorders can impair work ability at an earlier age, whereas musculoskeletal diseases most often arise from a degenerative pathogenesis at a later age. The findings are in line with the overall development of SA in Finland with musculoskeletal diseases being the most common reason for SA among older employees and mental disorders dominating among younger employees [6].

Our results support the previous knowledge on gender and socioeconomic differences in SA. Women were more likely to be assigned to either one of the trajectories with more SA in all of the models [2, 27]. Overall, women report poorer self-rated health [35] and are more active in seeking medical assistance, [36] which might lead to more medically certified SA spells.

In models examining all-cause and musculoskeletal disease–related SA, lower occupational class was associated with a higher likelihood of being assigned to either of the trajectories with SA. Highest occupational class might allow for better possibilities in modifying the work schedules during illness, greater control over personal workload, lower physical workload, or being able to work remotely. In a Swedish white collar worker cohort study on trajectories of long-term SA and disability pension, most employees belonged to trajectories with no or low SA and disability pension and employees reporting a low level of job control had a higher risk of belonging to a trajectory with SA or disability pension [37]. Previous studies have reported smaller differences between occupational classes in mental disorder–related SA than somatic diseases [38]. In line with this, we found an association only for ‘High MD SA’ with occupational class.

Shift work was associated with a higher likelihood of being assigned to trajectories with SA in the all-cause model when simultaneously adjusted for occupational class. This is in line with previous evidence that has shown associations between shift work and several health outcomes such as myocardial infarction and diabetes [39]. Shift work has been associated with poor mental health as well [40] but in our study we found no association between mental-health related SA and reporting shift work even when not adjusting for occupational class. We speculate, that healthy worker effect might have a role in the finding, if employees with mental health problems were selected to daytime work. Also, our data might be too small to identify possible differences in this regard.

Poor work—home satisfaction was associated with mental disorder–related SA ‘Low MD SA’ trajectory and with the all-cause ‘High SA’ trajectory, whereas no association was observed for musculoskeletal disease–related SA. This is in line with previous research that has found conclusive evidence between work family interface and mental health whereas evidence concerning physical health is scarce [41]. Common mental disorders such as depression affect an employee’s functional capacity, regardless of whether the symptoms arise from work- or family-related stressors. All in all, work arrangements and demands at work might have to be altered to help an employee with mental disorders to continue at work.

Those reporting shorter working hours were more likely to be assigned to the all-cause ‘High SA’ trajectory and mental disorder–related ‘High MD SA’ trajectory. We further analysed how working longer (over 40 h/week) versus shorter working hours was associated with trajectory membership (data not shown). Overtime work was more common in the ‘No SA’ (16%) than ‘High SA’ trajectory (11%). Long working hours have been associated with health problems [42] but in contrast to other European countries, the Nordic countries have shorter average working hours [43]. There are previous evidence supporting a negative association between long working hours and sickness absence [44.–45] Possible explanations for the finding include healthy worker effect and differences in working conditions and job motivation [44]. It is possible that the association between shorter working hours and high rate of SA is linked to possible lack of sufficient supportive workplace adaptations. Working hours might be reduced due to declining work ability or chronic conditions [43].

A previous person-based study showed that sleep problems and physical inactivity were associated with the high SA trajectory [9]. Similarly, variable oriented studies report associations between long-term SA and smoking, weight, and sleep problems [46]. Our results are in line with these studies: reporting smoking, overweight, or obesity was associated with trajectories with SA except for mental disorder ‘Low MD SA’ trajectory. In all three models, moderate or low leisure-time physical activity was associated with the most morbid trajectories. Those reporting sleep problems were more likely to belong to trajectories with SA. Binge drinking was associated with ‘High MD SA’ trajectory but negatively associated with ‘Low MSD. Overall, our results highlight the relevance of unhealthy health behaviour as a possible target for prevention also among ageing employees.

### Strengths and limitations of this study

Key strengths of this study include combining a large municipal cohort including a variety of self-report information, occupations, and work arrangements with register-based records of SA days and their diagnostic groups.

The trajectory approach has been suggested by previous studies as a potential tool to detect employees in need of support to maintain work ability. We utilised GBTM to identify SA patterns in a heterogeneous working population, instead of focusing on predetermined variables, with the upside of studying the population without prior assumptions.

We focused on employees. The healthy worker effect is hence an issue to be considered, meaning the possibility that people with more diminished work ability were selected out of the work force prior to our study [47, 48]. We also did not include those who continued to work from retirement.

Our study population was predominantly women in the municipal sector, hence, generalisation to the general working population should be done with caution. Municipalities are less sensitive to macroeconomic trends and one might argue that municipal employees take SA more easily than employees in the private sector, however, a study on the SA trajectory differences between municipalities and the private sector detected no clear difference [10]. We analysed medically confirmed long-term SA and did not have information shorter on SA spells, however, we were able to extract the specific diagnostic codes for the absence.

Information on health behaviours and work-related factors was based on self-report data, however, due to follow-up questionnaires, most of the participants had answered questions on health behaviour and working hours several times. Survey data do not allow for confirming causal relationships, but clear associations were observed.

A modified work description presents an alternative for SA, but still, few employees in the EU with disabilities have an adjusted workplace to accommodate their health [5]. With an ageing work force and a larger proportion of employees with chronic diseases, a consideration for individuality might be needed. Future studies should scrutinise the emerging subpopulations with SA and further examine the individual or environmental triggers leading to the possible cascade of increasing SA.

## Conclusions

We examined all-cause and diagnosis-specific trajectories of sickness absence in later career and identified varying socioeconomic and work- and health-related factors. Trajectories with a high rate of sickness absence were consistently associated with smoking, weight, leisure time physical inactivity, and sleep problems, indicating multiple health-related factors that can be taken into further consideration when designing interventions in occupational healthcare.

## Electronic supplementary material

Below is the link to the electronic supplementary material.

Supplementary Material 1

## Data Availability

The register data used for the study are not publicly shared due to the European data protection laws. Data can be requested from the corresponding author, Johanna Suur-Uski, upon a reasonable request.

## Abbreviations

SA: Sickness absence
GBTM: Group-based trajectory modelling
EU: The European Union
MD: Mental disorder
MSD: Musculoskeletal disease
SII: Social Insurance Institution of Finland
ICD-10: International classification of diseases, the tenth revision
BMI: Body mass index
MET: Metabolic equivalent
OR: Odds ratio
CI: Confidence interval
AIC: Akaike information criteria
BIC: Bayesian information criteria
