# Supplementary material for: Long-term sickness absence trajectories among ageing municipal employees – the contribution of social and health-related factors
Source: BMC Public Health. 2023 Jul 26;23:1429. doi: 10.1186/s12889-023-16345-9 (PMC10373243; doi:10.1186/s12889-023-16345-9)
Supplement: Supplementary file 1 — Supplementary Material 1 [file 12889_2023_16345_MOESM1_ESM.docx]

**Supplementary file**

**An example of code used in Stata in the all-cause SA model**

| **All-cause model** |
| --- |
| traj, var(SicknessAbsence*) indep(Time*) model(zip) order(1 1) iorder(0 1) dropout (2 2) detail star(strt) |

| Number of trajectories with chosen cause model | BIC, Sample-size adjusted BIC, AIC, Log likelihood, Entropy |
| --- | --- |
| 1 | BIC=-26279.15 (N=22260) BIC=-26272.87 (N=2747) AIC=-26255.11 ll= -26249.11, Entropy 1 |
| 2 | BIC=-25528.27 (N=22260) BIC=-25513.62 (N=2747) AIC=-25472.19 ll= -25458.19 Entropy 0,80, preference to this model due to BIC, entropy and clinical plausibility etc. |
| 3 | BIC=-25528.27 (N=22260) BIC=-25513.62 (N=2747) AIC=-25472.19 ll= -25458.19 Entropy 0.72 |
| 4 | BIC=-28760.48 (N=22260) BIC=-28736.42 (N=2747) AIC=-28668.36 ll= -28645.36 Entropy 0.80 |

**Supplementary tables:**

**Table 1** Characteristics of the study population (N=4729)

|  | The study population |  |
| --- | --- | --- |
| All | N=4729 | (%) |
| Gender |  |  |
| Women | 3778 | 80 |
| Men | 951 | 20 |
| Occupational class |  |  |
| Professionals and managers | 1525 | 32 |
| Semi-professionals | 1033 | 22 |
| Routine non manual workers | 1538 | 33 |
| Manual workers | 633 | 13 |
| Marital status |  |  |
| Cohabiting | 3313 | 70 |
| Non-cohabiting | 1415 | 30 |
| Work arrangements |  |  |
| Non-shift work | 3764 | 80 |
| Shift work | 961 | 20 |
| Working hours |  |  |
| Weekly mean (hours) | 34.7 |  |
| Work home satisfaction |  |  |
| Satisfied | 2590 | 56 |
| Not satisfied | 2059 | 44 |
| Smoking |  |  |
| Never or quit | 3775 | 80 |
| Daily or occasionally | 948 | 20 |
| Binge drinking (six or more units) | |  |
| Rarely or never | 4198 | 89 |
| Weekly or more often | 525 | 11 |
| Body mass index, BMI |  |  |
| Healthy weight (BMI<25) | 2310 | 49 |
| Overweight (25≤BMI<30) | 1618 | 34 |
| Obesity (BMI≥30) | 798 | 17 |
| Leisure-time physical activity |  |  |
| High | 1777 | 38 |
| Intermediate | 1888 | 40 |
| Low | 1040 | 22 |
| Sleep problems |  |  |
| No | 3788 | 81 |
| Yes | 908 | 19 |

**Table 2:** Characteristics of the all-cause long-term SA trajectories; SA=Sickness absence

|  | No SA | (%) | Low SA | (%) | High SA | (%) | P-value for chi2 |
| --- | --- | --- | --- | --- | --- | --- | --- |
| All | 1982 | 42 | 2183 | 46 | 564 | 12 |  |
| Gender |  |  |  |  |  |  |  |
| Women | 1499 | 76 | 1799 | 82 | 480 | 85 |  |
| Men | 483 | 24 | 384 | 18 | 84 | 15 | <0.01 |
| Occupational class |  |  |  |  |  |  |  |
| Professionals and managers | 815 | 41 | 606 | 28 | 104 | 18 |  |
| Semi-professionals | 455 | 23 | 461 | 21 | 117 | 21 |  |
| Routine non-manual workers | 528 | 27 | 785 | 36 | 225 | 40 |  |
| Manual workers | 184 | 9 | 331 | 15 | 118 | 21 | <0.01 |
| Marital status |  |  |  |  |  |  |  |
| Cohabiting | 1439 | 73 | 1497 | 69 | 377 | 67 |  |
| Non-cohabiting | 543 | 27 | 686 | 31 | 186 | 33 | <0.01 |
| Work arrangements |  |  |  |  |  |  |  |
| Non-shift work | 1660 | 84 | 1686 | 77 | 418 | 74 |  |
| Shift work | 320 | 16 | 496 | 23 | 145 | 26 | <0.01 |
| Working hours |  |  |  |  |  |  |  |
| Weekly mean (hours) | 34.9 |  | 34.5 |  | 34.3 |  | 0.2 |
| Work–home satisfaction |  |  |  |  |  |  |  |
| Satisfied | 1124 | 58 | 1196 | 56 | 270 | 49 |  |
| Not satisfied | 818 | 42 | 958 | 44 | 283 | 51 | <0.01 |
| Smoking |  |  |  |  |  |  |  |
| Never or quit | 1675 | 85 | 1707 | 78 | 485 | 70 |  |
| Daily or occasionally | 304 | 15 | 473 | 22 | 79 | 30 | <0.01 |
| Binge drinking (six or more units) | |  |  |  |  |  |  |
| Rarely or never | 1768 | 89 | 1945 | 89 | 714 | 86 |  |
| Weekly or more often | 209 | 11 | 237 | 11 | 108 | 14 | 0.07 |
| Body mass index, BMI |  |  |  |  |  |  |  |
| Healthy weight (BMI<25) | 1067 | 54 | 1027 | 47 | 216 | 38 |  |
| Overweight (25≤ BMI<30) | 650 | 33 | 752 | 34 | 216 | 14 |  |
| Obesity (BMI≥30) | 264 | 13 | 402 | 18 | 132 | 23 | <0.01 |
| Leisure-time physical activity |  |  |  |  |  |  |  |
| High | 839 | 43 | 798 | 37 | 140 | 25 |  |
| Intermediate | 723 | 37 | 916 | 42 | 249 | 44 |  |
| Low | 409 | 21 | 459 | 21 | 172 | 31 | <0.01 |
| Sleep problems |  |  |  |  |  |  |  |
| No | 1660 | 84 | 1736 | 80 | 392 | 71 |  |
| Yes | 308 | 16 | 436 | 20 | 164 | 29 | <0.01 |

**Table 3:** Characteristics of mental disorder–related long-term sickness absence (SA) trajectories; MD=Mental disorder

|  | No MD SA | (%) | Low MD SA | (%) | High MD SA | (%) | P-value for chi2 |
| --- | --- | --- | --- | --- | --- | --- | --- |
| All | 4032 | 85 | 482 | 10 | 215 | 5 |  |
| Gender |  |  |  |  |  |  |  |
| Women | 3171 | 79 | 427 | 89 | 180 | 84 |  |
| Men | 861 | 21 | 55 | 11 | 25 | 16 | <0.01 |
| Occupational class |  |  |  |  |  |  |  |
| Professionals and managers | 1338 | 33 | 146 | 30 | 41 | 19 |  |
| Semi-professionals | 886 | 22 | 97 | 20 | 50 | 23 |  |
| Routine non-manual workers | 1272 | 32 | 179 | 37 | 87 | 40 |  |
| Manual workers | 536 | 13 | 60 | 12 | 37 | 17 | <0.01 |
| Marital status |  |  |  |  |  |  |  |
| Cohabiting | 2869 | 71 | 301 | 62 | 143 | 67 |  |
| Non-cohabiting | 1162 | 29 | 181 | 38 | 72 | 33 | <0.01 |
| Work arrangements |  |  |  |  |  |  |  |
| Non-shift work | 3222 | 80 | 379 | 79 | 163 | 76 |  |
| Shift work | 807 | 20 | 103 | 21 | 51 | 24 | 0.34 |
| Working hours |  |  |  |  |  |  |  |
| Weekly mean (hours) | 34.7 |  | 34.3 |  | 33.9 |  | 0.56 |
| Work–home satisfaction |  |  |  |  |  |  |  |
| Satisfied | 2262 | 57 | 226 | 47 | 102 | 48 |  |
| Not satisfied | 1700 | 43 | 250 | 53 | 109 | 52 | <0.01 |
| Smoking |  |  |  |  |  |  |  |
| Never or quit | 3253 | 81 | 374 | 78 | 148 | 69 |  |
| Daily or occasionally | 774 | 19 | 107 | 22 | 67 | 31 | <0.01 |
| Binge drinking (six or more units) |  |  |  |  |  |  |  |
| Rarely or never | 3589 | 89 | 423 | 88 | 186 | 87 |  |
| Weekly or more often | 437 | 11 | 59 | 12 | 29 | 13 | 0.35 |
| Body mass index, BMI |  |  |  |  |  |  |  |
| Healthy weight (BMI<25) | 1998 | 50 | 236 | 49 | 76 | 35 |  |
| Overweight (25≤ BMI<30) | 1377 | 34 | 158 | 33 | 83 | 39 |  |
| Obesity (BMI≥30) | 655 | 16 | 87 | 18 | 56 | 26 | <0.01 |
| Leisure-time physical activity |  |  |  |  |  |  |  |
| High | 1564 | 39 | 166 | 35 | 47 | 22 |  |
| Intermediate | 1597 | 40 | 197 | 41 | 94 | 44 |  |
| Low | 849 | 21 | 118 | 25 | 73 | 34 | <0.01 |
| Sleep problems |  |  |  |  |  |  |  |
| No | 3297 | 82 | 351 | 73 | 140 | 66 |  |
| Yes | 706 | 18 | 129 | 27 | 73 | 34 | 0.25 |

**Table 4:** Characteristics of musculoskeletal disease–related long-term sickness absence (SA) trajectories; MSD=Musculoskeletal disease

|  | No MSD SA | (%) | Low MSD SA | (%) | High MSD SA | (%) | P-value for chi2 |
| --- | --- | --- | --- | --- | --- | --- | --- |
| All | 3456 | 73 | 930 | 20 | 343 | 7 |  |
| Gender |  |  |  |  |  |  |  |
| Women | 2699 | 78 | 773 | 83 | 306 | 89 |  |
| Men | 757 | 22 | 157 | 17 | 37 | 11 | <0.01 |
| Occupational class |  |  |  |  |  |  |  |
| Professionals and managers | 1294 | 37 | 179 | 19 | 52 | 15 |  |
| Semi-professionals | 801 | 23 | 171 | 18 | 61 | 18 |  |
| Routine non-manual workers | 992 | 29 | 387 | 42 | 159 | 46 |  |
| Manual workers | 369 | 11 | 193 | 21 | 71 | 21 | <0.01 |
| Marital status |  |  |  |  |  |  |  |
| Cohabiting | 2447 | 71 | 638 | 69 | 228 | 66 |  |
| Non-cohabiting | 1008 | 29 | 292 | 31 | 115 | 34 | 0.13 |
| Work arrangements |  |  |  |  |  |  |  |
| Non-shift work | 2835 | 82 | 676 | 73 | 253 | 74 |  |
| Shift work | 618 | 18 | 253 | 27 | 90 | 26 | <0.01 |
| Working hours |  |  |  |  |  |  |  |
| Weekly mean (hours) | 34.8 |  | 34.4 |  | 34.3 |  | <0.01 |
| Work–home satisfaction |  |  |  |  |  |  |  |
| Satisfied | 1895 | 56 | 530 | 58 | 165 | 49 |  |
| Not satisfied | 1499 | 44 | 388 | 42 | 172 | 51 | 0.02 |
| Smoking |  |  |  |  |  |  |  |
| Never or quit | 2859 | 83 | 682 | 73 | 234 | 68 |  |
| Daily or occasionally | 593 | 17 | 246 | 27 | 109 | 32 | <0.01 |
| Binge drinking (six or more units) |  |  |  |  |  |  |  |
| Rarely or never | 3061 | 89 | 839 | 90 | 298 | 87 |  |
| Weekly or more often | 390 | 11 | 90 | 10 | 45 | 13 | 0.18 |
| Body mass index, BMI |  |  |  |  |  |  |  |
| Healthy weight (BMI<25) | 1786 | 52 | 401 | 43 | 123 | 36 |  |
| Overweight (25≤ BMI<30) | 1155 | 33 | 328 | 35 | 135 | 39 |  |
| Obesity (BMI≥30) | 514 | 15 | 199 | 21 | 85 | 25 | <0.01 |
| Leisure-time physical activity |  |  |  |  |  |  |  |
| High | 1395 | 41 | 309 | 33 | 73 | 21 |  |
| Intermediate | 1319 | 38 | 409 | 44 | 160 | 47 |  |
| Low | 726 | 21 | 206 | 23 | 108 | 32 | <0.01 |
| Sleep problems |  |  |  |  |  |  |  |
| No | 2823 | 82 | 737 | 80 | 228 | 68 |  |
| Yes | 611 | 18 | 188 | 20 | 109 | 32 | <0.01 |

**Supplementary figures:**

Individual lines plotted by trajectory group.

Figure 1: All-cause SA individual trajectories, group 1 = ‘High SA’, group 2 = ‘Low SA’

Figure 2: Drop-out probability of SA trajectories from ages 50 to 60. All-cause SA group 1 = ‘High SA’, group 2 = ‘Low SA’

Figure 3: Mental disorder–related individual SA trajectories, group 1 = ‘High MD SA’, group 2 = ‘Low MD SA’

Figure 4: Drop-out probability of Mental disorder–related SA trajectories from ages 50 to 60. All-cause SA group 1 = ‘High SA’, group 2 = ‘Low SA’

Figure 5: Musculoskeletal disease–related individual SA trajectories, group 1 = ‘High MSD SA’, group 2 = ‘Low MSD SA’

Figure 6: Drop-out probability of Musculoskeletal disease–related SA trajectories from ages 50 to 60. Musculoskeletal disease-related SA, group 1 = ‘High MSD SA’, group 2 = ‘Low MSD SA’
